# Cardiac Glucolipotoxicity and Cardiovascular Outcomes

**DOI:** 10.3390/medicina54050070

**Published:** 2018-10-11

**Authors:** Marlon E. Cerf

**Affiliations:** 1 Biomedical Research and Innovation Platform, South African Medical Research Council, Tygerberg 7505, South Africa; marlon.cerf@mrc.ac.za; Tel.: +27-21-938-0818; 2 Division of Medical Physiology, Department of Biomedical Sciences, Faculty of Medicine and Health Sciences, University of Stellenbosch, Tygerberg 7505, South Africa

**Keywords:** bioenergetics, cardiac development, diabetes, fatty acids, glucose transport, hyperglycemia

## Abstract

Cardiac insulin signaling can be impaired due to the altered fatty acid metabolism to induce insulin resistance. In diabetes and insulin resistance, the metabolic, structural and ultimately functional alterations in the heart and vasculature culminate in diabetic cardiomyopathy, coronary artery disease, ischemia and eventually heart failure. Glucolipotoxicity describes the combined, often synergistic, adverse effects of elevated glucose and free fatty acid concentrations on heart structure, function, and survival. The quality of fatty acid shapes the cardiac structure and function, often influencing survival. A healthy fatty acid balance is therefore critical for maintaining cardiac integrity and function.

## 1. Introduction

Glucotoxicity was first described in the islets and defined as non-physiological and potentially permanent β-cell damage due to persistent hyperglycemia [1] but it is also implicated in the pathogenesis of cardiovascular disease (CVD). Chronic hyperglycemia modulates the expression of multiple key glucotoxic proteins [2] thereby disrupting intracellular signal transduction by altering kinase C protein activity, generating reactive oxygen species (ROS), activating endoplasmic reticulum (ER)-stress, generating advanced glycation end products (AGEs), activating the polyol pathway and hexosamines, and increasing proinflammatory cytokine and growth factor release [2,3,4]. These metabolic derangements contribute to endothelial dysfunction that may progress to atherosclerosis [2,3,4].

Fatty acids (FAs) are essential substrates for energy production, and individual FAs induce various multiple signaling pathways for cellular function [5]. FA overload may damage the myocardium through high FA β-oxidation and toxic lipid species accretion in the myocardium [5] presenting lipotoxicity [6]. Excess lipids, or their metabolites and derivatives, in organs, i.e., lipotoxicity, impair insulin signaling and activate hepatic, skeletal muscle and cardiac inflammation. Insulin resistance is intrinsically linked to a dysregulated FA metabolism [7].

Glucolipotoxicity refers to the combined, often synergistic deleterious actions of elevated glucose and free fatty acid (FFA) concentrations on β-cell physiology and survival [8,9,10] that can be extended to other organs such as the heart. Obesity prompts inflammation, insulin resistance, hyperleptinemia, elevated circulating FFA and triglyceride concentrations, and hypertension [11]. These compromised metabolic states, in conjunction with a high saturated fat diet and high glycemic load, elicit deleterious cardiac effects characterized by the myocardial accumulation of toxic glucose and lipid intermediates [11]. Further, these metabolic derangements can trigger cardiomyocyte apoptosis, fibrosis, cardiomyocyte hypertrophy, mitochondrial dysfunction, and impaired systolic and diastolic function that ultimately lead to heart failure [11]. Hence, glucolipotoxicity compromises cardiac structure and function that may induce CVD. Therefore, physiological glucose and lipid concentrations are required to maintain cardiac integrity and function.

## 2. Cardiac Development, Bioenergetics, and Physiology

During fetal development, the heart, pancreas, and liver are highly sensitive to maternal body composition fluctuations prior to conception and/or during early gestation. Maternal obesity and dysregulated metabolism induce fetal visceral fat deposition, thereby increasing the offspring’s susceptibility to metabolic disease later in life [12]. Overall, the degree of pre-gestation maternal overfeeding and adiposity determine the fetal overgrowth trajectory and mid-gestational fetal organ development, with milder maternal adiposity inducing minor changes [13].

Adult hearts have a low regenerative potential [14,15], therefore, the loss of cardiac muscle through ischemic injury (myocardial infarction) far exceeds the cardiac regenerative capacity for new cardiomyocytes [16]. The subsequent cardiomyocyte deficit, ~one billion cells [16], markedly weakens cardiac pump function that progresses to cardiac failure [17]. Therefore, it is critical that heart development is optimal to provide the structure to enable function, particularly upon metabolic challenges later in life.

Early heart growth is accompanied by mononucleated cardiomyocyte proliferation [18]. By mid-gestation, these mononucleated cardiomyocytes develop into binucleated cardiomyocytes thereby enhancing cardiac mass by hypertrophy [19,20]. In humans and sheep, adult cardiomyocyte endowment is largely determined prior to birth [21]. Positive influences to enable optimal heart development and growth during fetal life is critical. Any insult, such as high-fat feeding with exposure to excess harmful FAs, such as saturated FAs, and glucose, may impair fetal heart development that is exacerbated by maternal obesity.

The heart has high energy demands. In fetal hearts, glucose and lactate are the primary energy sources, whereas postnatally, FA β-oxidation provides energy [22,23,24]. Gestational diabetes, persistent hyperglycemia, and hyperlipidemia all contribute to shaping heart development, structure, and function. Glucose is the primary energy source for fetal cardiomyocytes likely attributed to limited circulating FA availability or immature enzyme systems [18]. Cardiomyocyte maturation may be linked to cardiac metabolism but is dependent on several factors that influence cardiac maturation and metabolism [18]. Glucocorticoids are required in late gestation for fetal organ system maturation such as the heart, lung, and gut [25]. The fetal cardiac glucose uptake is facilitated through the insulin-independent glucose transporter type 1 (GLUT1) [26] with anaerobic glycolysis largely meeting the ATP demands [27]. However, with no continuous placental nutrient supply shortly after birth, the circulating oxygen supply rapidly increases to drive mitochondrial ATP production to maintain cardiac energy demands over the life course [28]. These events coincide with the shift to FA β-oxidation as the dominant ATP provider in normal resting adult hearts [23].

Human hearts have high-energy demands with a daily ATP turnover of ~30 kg [29]. Thus, the heart requires a high oxygen uptake to generate sufficient ATP by oxidative phosphorylation for proper function [30]. Cardiac diseases are characterized by an altered myocardial energy metabolism [30]. A general decrease in oxidative capacity and down-regulation of FA β-oxidation enzymes in cardiac muscle were observed in heart failure [28,31,32,33,34,35]. In diabetic patients, mitochondria in the heart had decreased FA-supported respiratory capacity and a reduced capacity to oxidize palmitoylcarnitine [36]. This was confirmed in the mitochondria of obese and diabetic mice with reduced oxidative capacity [37]. Further, there is a reduction in both FA β-oxidation genes and enzyme expression profiles, and reduced myocardial energy generation in human and animal heart failure [31,32,33,34,35]. In addition, insulin resistance may be associated with incomplete FA β-oxidation, thereby yielding an increase in acylcarnitines [38]. In primary myotubes, a reduction of short-chain acylcarnitine and acetylcarnitine formation was reported to protect against palmitate-induced insulin resistance [38]. The decrease in FA-supported respiration may result in an increase in incomplete FA β-oxidation and the subsequent increase in short-chain acylcarnitines, likely linked to the onset of insulin resistance [30].

In humans and rats, an increase in dietary fat saturation contributed to higher systolic and diastolic blood pressure [39,40]. In rats, high omega-6 FA diets have antihypertensive effects on angiotensin (Ang) II-induced hypertension [41]. FA quality, therefore, influences cardiac function. An Ang II-induced increase in left ventricular c-fos mRNA expression was higher in high-fat diet (HFD) fed rats fed but not altered in polyunsaturated FA fed rats [42]. The quality of dietary fat alters early hypertrophic gene activation in the pressure-overloaded myocardium and requires the activation of the activator protein 1 (AP1) and mitogen-activated protein kinases (MAPK) signal transduction pathways [43]. The quality of fat also shapes the cardiac structure through activating and altering cardiac-specific factors. A diet with an adequate mix of FAs, high in beneficial FAs and low in harmful FAs, is required for maintaining cardiac health.

Adult diabetic cardiomyopathy may involve an irregular cardiac metabolism and subsequently lead to oxidative stress, mitochondrial dysfunction, and mitophagy [44,45,46,47]. Cellular bioenergetics play increasingly key roles in maintaining health and in the pathogenesis of disease [48], particularly the heart [49]. Due to hearts’ high-energy requirements, a continuous fuel supply is required to sustain perpetual contractile activity, thereby rendering it susceptible to failure in the presence of any metabolic and/or mitochondrial derangements [28]. In adults, the normal resting hearts’ energy demands are primarily sustained by FAs [23,46,50,51]. Despite a FA preference, the hearts can use different substrates dependent on supply and demand [12]. This fuel flexibility enables continuous energy production under varying metabolic states such as developmental maturation, starvation (fuel supply), exercise (fuel demand), and ischemia (oxygen supply) [28,50,52]. Diminished fuel flexibility from excess circulating fuels, such as glucose and FAs, and compromised metabolic states, such as insulin resistance, predisposes to heart failure [46,49].

The adverse cardiovascular effects of obesity (such as cardiomyocyte FA overload) likely encompasses arrhythmias, cardiomyopathy, and heart failure [53,54]. The normal heart attains ≥60% of its energy requirements from long-chain FA β-oxidation to yield ATP [55]. Cardiac hypertrophy is linked to the suppression of myocardial FA β-oxidation with the reversion of increased glucose utilization [55,56], suggesting an association of altered myocardial FA metabolism and cardiac hypertrophy. In neonatal ventricular cardiomyocytes, long-chain FAs were observed to alter Ang II-induced hypertrophic responses [57]. In vivo, prolonged dietary FA intake also influences the progression to left ventricular hypertrophy [58,59,60,61,62], which reflects cardiac remodeling in response to the FA intake.

## 3. Postnatal Cardiac Insulin Signaling and Insulin Resistance

Postnatally, GLUT4 regulates the cardiac glucose uptake through the insulin signaling pathway viz., the insulin receptor (IR), insulin receptor substrate-1 (IRS1), phosphatidylinositol 3-kinase (PI3K), 3-phosphoinositide-dependent protein kinase 1 (PDPK1) and/or Akt [18] although different insulin signaling factor isoforms and types may be activated to achieve cardiac glucose uptake. PDPK1 activation induces atypical protein kinase C zeta (PKCζ) phosphorylation and activation, whereas Akt phosphorylation induces Akt substrate 160 kDa (AS160) phosphorylation and activation [18]. Phosphorylated PKCζ and AS160 are both integral for GLUT4 translocation to plasma membranes for glucose uptake [63].

Despite insulin resistance being a great predictor for CVD, it is rarely the sole contributor to the disease in patients [64]. In diseased states, such as diabetes and insulin resistance, the metabolic, structural and ultimately functional alterations in the heart and vasculature culminate in diabetic cardiomyopathy, coronary artery disease, ischemia and eventual heart failure [65,66]. The impairment of insulin-stimulated glucose uptake is the first and steadiest alteration that occurs in the hearts of animal models in the insulin resistance evolution [67]. This change occurs prior to defects in insulin’s capacity to stimulate or elevate PI3K/Akt signaling, and is attributed to the GLUT4 protein reduction in combination with an impairment of GLUT4 membrane translocation.

The development of hyperinsulinemia and insulin resistance in murine cardiac hypertrophy is due to the pressure overload boosts in myocardial insulin signaling to Akt (in excess) which adds to the left ventricular reconstruction at an accelerated level and ultimately, a shift to heart failure [68]. The heart responds to insulin, and insulin resistance is a prominent defect in individuals who suffer from diabetes, obesity and metabolic syndrome [69,70]. HF feeding often triggers obesity, insulin resistance, metabolic syndrome, diabetes, and CVD. In mice, HFD-induced myocardial insulin resistance was established within ten days [71]. There was also an association between insulin resistance and decreased glucose uptake in the myocardium, reduced PKB/Akt activity and reduced GLUT4 levels that preceded and was independent of systemic insulin resistance [71].

In myocardial insulin resistance, the rate of FA β-oxidation remains normal or may be increased, but the rate of glucose oxidation is usually decreased whether insulin-stimulated or non-insulin-stimulated [72]. ROS are free radicals and by-products of reduction-oxidation reactions under physiological conditions in eukaryotic cells [73]. Progeny from nutrient-limited rat dams developed hypertension concomitant with elevated oxidative stress in mesenteric arterioles. Further, apart from vascular dysfunction, intrauterine growth restriction (IUGR) promoted ROS-mediated cardiac damage [74]. An increase in the lipid uptake and its subsequent oxidation, e.g., as in insulin resistance, can give rise to cellular lipid intermediate accumulation, excess mitochondrial or peroxisome ROS production, and cardiac derangements, leading to dysfunction [72]. This was demonstrated by the over-expression of cardiac-specific peroxisome proliferator-activated receptor alpha (PPARα) that induced increased cardiac lipid oxidation, deranged metabolism and subsequently led to both structural and functional alterations detrimental to the heart [65]. The induction of insulin resistance in mice by exposure to a HFD also triggered the reconstruction of the heart and systolic dysfunction [71]. The heart’s ability to tolerate and withstand ischemia and reperfusion can be constrained by myocardial insulin resistance by reducing the glucose uptake as well as the synthesis of glycogen and glycolysis, all which contribute to ATP delivery in the ischemic heart for cellular metabolism [75].

## 4. Postnatal Cardiac Fatty Acid Signaling

Cardiac FA uptake is facilitated by fatty acid translocase (FAT/CD36) and fatty acid transport protein 1 (FATP1) [76]. However, FA β-oxidation is regulated by adiponectin receptor 1 (AdipoR1) activation through adiponectin binding, leading to AMP-activated protein kinase (AMPK) phosphorylation and activation that subsequently phosphorylates acetyl CoA carboxylase (ACC) [77,78]. ACC catalyzes malonyl CoA production, thereby inhibiting carnitine palmitoyltransferase-1 (CPT1) from enabling FA transport into the mitochondria [79]. Peroxisome proliferator-activated receptor gamma coactivator 1 alpha (PGC1α) and peroxisome proliferator-activated receptor alpha (PPARα) both also regulate cardiac FA β-oxidation, thereby stimulating mitochondrial biogenesis and FA β-oxidation via increased transcription of regulators such as CPT1 [80]. Pyruvate dehydrogenase kinase-4 (PDK4) helps promote cardiac FA β-oxidation through glucose oxidation inhibition (via pyruvate dehydrogenase complex inhibition) [81]. However, during late gestation in fetal sheep, the activation of the insulin growth factor 2 receptor (IGF2R) signaling induces cardiac hypertrophy [82], evident by the atrial natriuretic peptide (ANP) overexpression [83].

Cardiac bioenergetics differs during fetal and postnatal life (Table 1). In the fetal heart, glucose transport through GLUT1 is insulin-independent and meets the ATP requirements through anaerobic glycolysis. In the developed heart, FAs primarily meet the heart’s energy requirements through FA β-oxidation. Should an alternative fuel source be required in states of high energy demands or low FA availability, insulin-dependent glucose is transported into the heart through GLUT4. In the developed heart, cardiac FA uptake is facilitated by FAT/CD36 and FATP1, with both FA transporters also localized in the fetal heart. In the fetal heart, although insulin signaling may be altered due to insults (e.g., high-fat programming where the heart is exposed to excess FAs during development), overt insulin resistance does not present but may manifest later in life. In the developed heart with cardiac insulin resistance, the rate of glucose oxidation is usually decreased (independent of insulin-stimulation), whereas the rate of FA β-oxidation remains normal or may be increased.

## 5. High Fat Diets and Fatty Acids Alter Cardiac Gene Expression Involved in Hypertrophy

Several inherited FA metabolism disorders are characterized by cardiac hypertrophy and cardiomyopathy [84]. Given that FAs are the mature heart’s primary energy source and that decreased FA utilization is implicated in the onset of cardiac hypertrophy [55], dietary FA supplementation may impact early hypertrophic processes and activate cardiac genes expressed during pressure overload [43]. This was reported in a study where excess feeding of saturated or polyunsaturated FAs was shown to distinctly modify the expression of the hypertrophy-associated genes ANP, B-type natriuretic peptide (BNP), and skeletal α-actin in response to Ang II-induced pressure overload [43]. In rats maintained on high linoleic acid (an omega-6 FA), Ang II exerted a marked increase in ANP, BNP, and skeletal α-actin mRNA expression relative to maintenance on a high saturated fat diet [43]. Thus, different fat classes elicit variable effects on cardiac gene expression that influence cardiac hypertrophy that may also be dependent on the timing and duration of the dietary administration.

## 6. Cardiac Glucolipotoxicity

### 6.1. Cardiac Bioenergetics in Cardiovascular Disease

The metabolic milieu informs the cardiac metabolic adaptation for the conversion of energy from many oxidizable substrates to sustain contractile energy needs [11]. In heart failure, the ATP turnover rate reduces, whereas the substrate uptake rate may not reduce proportionally [11]. Thus, an imbalance in cell-substrate availability and intracellular substrate oxidation with the ensuing accumulation of intermediates derived from glucose and FA metabolism presents a vicious cycle [11]. When FA uptake surpasses fat oxidation capacity, the lipid overload prompts an increase in the triglyceride storage and lipid metabolite accumulation (especially ceramides), concomitant with higher oxidative and ER stress [85], and lipid-induced membrane function modifications [11].

### 6.2. Diabetes and Cardiovascular Disease

Diabetes and CVD are intrinsically linked. Diabetes increases CVD risk, exacerbated by coexistent hypertension [86]. Causal mechanisms that induce microvascular and macrovascular diabetic complications such as oxidative stress, inflammation, and fibrosis, also induce vascular remodeling and dysfunction in hypertension [86]. In diabetes and hypertension, augmented vascular oxidative stress induces post-translational oxidative protein alterations prompting cellular damage and vascular dysfunction [86]. In diabetic and obese adults, elevated circulating glucose and FA concentrations, and compromised insulin activity may contribute to diabetic cardiomyopathy [12]. In adult diabetic cardiomyopathy, first diastolic then systolic dysfunction, cardiac hypertrophy, and heart failure are often present without the influence of additional cardiovascular risk factors [44,45]. In some obese diabetic patients, cardiomyopathy presents as characterized by contractile dysfunction specific to cardiac muscle [87,88,89,90]. In obese and diabetic rodents, the onset of cardiomyopathy includes diastolic dysfunction [91,92], cardiomyocyte hypertrophy [93,94,95], interstitial fibrosis [93,96,97], early-onset metabolic maladaptation [94,95,98,99], and progressive lipid accumulation [94,95], all precursors to heart failure [100]. Cardiomyopathy is typically accompanied by metabolic inflexibility attributed to an insulin deficiency or impaired action thereby causing hyperglycemia; hypergycemia with the overconsumption of harmful FAs, represent precursors to glucolipotoxicity [11,87,101,102].

### 6.3. The Obesity Paradox

Some obese individuals do not present with CVD. Although obesity increases CVD [103], obese patients who recover from heart failure and acute myocardial infarction have improved survival outcomes which describes the obesity paradox [104]. The obesity paradox is influenced by comorbidities, confounders, selection (or survival) bias, and the utilization of body mass index (BMI) for weight classification [103]. Comorbidities include CVD and diabetes. For confounding factors, smoking is linked to reduced BMI and increased mortality [103]. Selection bias arises in obese patients with an earlier onset of disease or untimely death which excludes them from studies [103]. BMI does not account for body composition, fat distribution, fat and lean mass, all factors that may influence CVD risk [103]. Waist circumference combined with BMI is a better proxy for identifying obese individuals predisposed to CVD [103]. The obesity paradox is complex and requires further elucidation. However, when obese individuals remain in their compromised metabolic state, which is exacerbated by the persistent consumption of unhealthy diets and leading inactive lifestyles, they become more susceptible to CVD.

### 6.4. Cardiac Lipotoxicity and Cardioprotection

The quality of fat determines shifts to cardiac lipotoxicity or cardioprotection. For example, excess saturated FA intake would impose cardiac lipotoxicity whereas optimal omega-3 FA intake would confer cardioprotection. A high saturated fat diet has adverse effects on cardiovascular health. Dietary saturated FAs, compared to polyunsaturated FAs such as omega-3 FAs, increase circulating low-density lipoprotein cholesterol (LDL-C), which enhances the susceptibility to coronary heart disease [105]. Further, specific saturated FAs variably modify plasma lipoprotein concentrations, with each saturated FA (excluding stearic acid) inducing an increase in LDL- and high-density lipoprotein cholesterol (HDL-C) concentrations with a reduction in triglyceride concentrations [105]. Dietary saturated FA intake should be <10% of the total caloric intake [106] as reducing dietary saturated FAs lowered cardiovascular events by 17% [107] with each 5% increase in the saturated FA intake linked to an 8% increase in the risk for mortality [108]. Replacing saturated FAs with polyunsaturated FAs, such as omega-3 FAs, lowers the mortality risk from CVD [108]. High omega-3 FA consumption, especially docosahexaenoic acid and eicosaehexanoic acid, protects against CVD through decreasing circulating triglyceride concentrations, inflammation, and lethal arrhythmias, also enhancing mitochondrial function and remodeling membranal phospholipids [109]. Therefore, beneficial omega-3 FAs should be considered to replace saturated FAs, the latter which are harmful in excess, to confer some cardioprotection in patients with CVD.

### 6.5. Gluco-, Lipo- and Glucolipotoxicity

Persistent hyperglycemia presents glucotoxicity whereas continuous exposure to harmful FAs presents lipotoxicity. Where both glucotoxicity and lipotoxicity coexist, often in obesity, insulin resistance, diabetes and CVD, glucolipotoxicity often triggers synergistic adverse metabolic outcomes. Chronic overconsumption of fat and carbohydrates (e.g., glucose) elevates circulating insulin, leptin, FFA and triglyceride concentrations, with or without diabetes, thereby exposing the heart to toxic anabolic stimuli and an overabundance of oxidative substrates [11]. Chronic increased insulin demand, e.g., obesity, insulin resistance, β-cell dysfunction and diabetes, predisposes to the onset of metabolic disease [104] that includes adverse cardiac outcomes and CVD. Chronic HF feeding is linked to the etiology of obesity, insulin resistance, β-cell dysfunction [104], diabetes and CVD. A constant increase in FFA or saturated FA intake from a HFD may trigger adipogenesis thereby contributing to obesity, an altered inflammatory response, insulin resistance, metabolic syndrome [105,106], diabetes and CVD. Therefore, there is a close association between diabetes and CVD through HF consumption, i.e., increased harmful FA intake (e.g., saturated FAs) and hyperglycemia through glucolipotoxicity.

There is consensus that prolonged cardiac exposure to excess circulating fuels (e.g., glucose, FFAs, and triglycerides) and growth hormones (e.g., insulin and leptin) speed up myocardial pathology that leads to cardiac hypertrophy and failure [11]. Both elevated circulating glucose and FFAs have deleterious effects on health outcomes through glucolipotoxicity. Tissue-specific cellular damage, induced by ROS, has been observed with excess glucose and FFA exposure [107,108,109], resulting in glucolipotoxicity-induced oxidative stress, a driver of apoptosis [110]. There is elevated oxidative stress in uncontrolled diabetes and potentially in the evolution of β-cell and cardiomyocyte dysfunction in diabetes [111,112,113]. High glucose in combination with the saturated FA, palmitic acid, consistently caused cardiomyoblast apoptosis [110]. In vitro, hyperglycemia and high palmitic acid exposure induced apoptosis and increased intracellular ROS levels in rat heart myoblast cells (H9c2), suggesting that glucolipotoxicity-induced cardiomyocyte apoptosis was triggered by intracellular ROS generation [110]. The heart’s impaired ability to oxidize fat and carbohydrates prompts non-oxidative metabolic intermediate and ROS accumulation [71]. In humans, this dysregulated fuel metabolism also presents in obesity, diabetes, and heart failure [114].

Cardiomyocyte glucolipotoxicity primarily originates or terminates in the mitochondria and the ER [112,115,116,117,118]. In cardiomyocyte glucolipotoxicity, protein quality control is impaired, resulting in ER stress and activation of the protein degradation pathway [119,120]. Chronic glucolipotoxicity aggravates ER stress, saturates and impairs proteasomal protein degradation, thereby resulting in toxic misfolded protein accumulation [121,122]. In obesity and diabetes, glucolipotoxicity precedes diabetic cardiomyopathy [95,96,99,100,114], inferring that cardiac glucolipotoxicity may induce cardiomyocyte dysfunction [101]. Further, in obese diabetic hearts, glucolipotoxicity limits autophagy and macroautophagy, linked to insufficient transcriptional regulation by transcription factor EB (TFEB) [101]. The TFEB content loss through glucolipotoxicity may be attributed to increased TFEB inhibitory phosphorylation thereby preventing TFEB nuclear translocation to activate coordinated lysosomal enhancement and regulation (CLEAR) genes for lysosomal autophagic and functional maintenance [101]. The inhibition of TFEB impairs the lysosome function causing proteotoxicity, ER stress, mitochondrial dysfunction, and cell death thereby predisposing obese diabetic hearts to cardiomyopathic failure [101]. Saturated FAs alone influence TFEB regulation, exacerbated by elevated glycemia [101] reflecting glucolipotoxicity. Palmitate myocyte overloading (but not oleate) diminished cellular TFEB and impaired autophagy, inferring that TFEB regulation and cardiomyocyte autophagy is dependent on the FA type [101]. Glucolipotoxicity-induced TFEB content reduction was also observed in class 1 obese patients’ hearts [101]. Thus, with glucolipotoxicity, lysosomal autophagic suppression was linked to a decreased lysosomal content, reduced activity of cathepsin-B, and decreased cellular TFEB content, thereby increasing the myocytes’ vulnerability to cardiac injury [101].

### 6.6. Glucolipotoxicity Shapes Cardiac Outcomes

**Figure 1:**
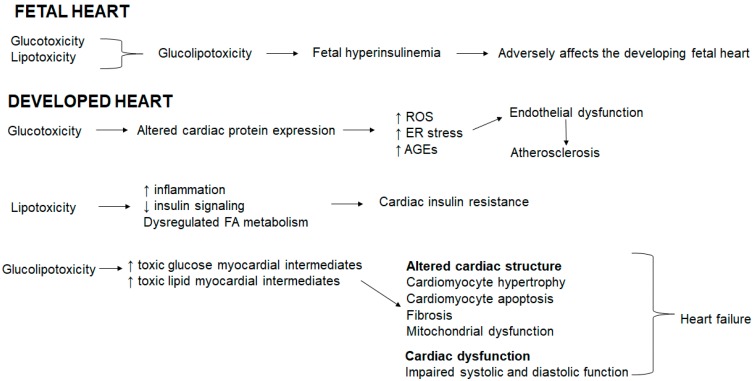
Glucolipotoxicity shapes cardiac outcomes.

In the fetal heart, glucotoxicity and lipotoxicity coexist resulting in glucolipotoxicity that induces fetal hyperinsulinemia [12]. Fetal hyperinsulinemia adversely affects the developing fetal heart (Figure 1). In the developed heart, glucotoxicity alters the cardiac protein expression, resulting in an increase in ROS, ER stress and advanced glycation end products (AGEs) that triggers endothelial dysfunction and atherosclerosis (Figure 1). Hyperglycemia accelerates AGE formation, AGEs accumulate in blood vessels and subsequently induce cardiovascular damage triggered in advanced diabetes [123]. AGEs stimulate ROS generation, which in turn further enhances AGE formation [123]. In addition, hyperglycemia prompts redox-sensitive protein kinase C, polyol and hexosamine pathway activation, thereby exacerbating mitochondrial dysfunction, oxidative stress, ER stress, and cellular damage [124,125,126,127,128]. Oxidative stress is linked to reduced nitric oxide (a vasodilator) bioavailability thereby inducing endothelial dysfunction [86]. Lipotoxicity increases inflammation, reduces insulin signaling and dysregulates the FA metabolism resulting in cardiac insulin resistance (Figure 1). Glucolipotoxicity increases both toxic glucose and lipid myocardial intermediates which collectively alters the cardiac structure evident by cardiomyocyte hypertrophy and apoptosis, and fibrosis concomitant with mitochondrial dysfunction and compromised the systolic and diastolic function, which ultimately precede heart failure (Figure 1).

## 7. Future Directions

Future research should focus on defining the proper healthy FA balance throughout different life stages and different states of metabolic disease to support a healthy heart. For example, glucose is the main energy supplier for the developing heart and essential FAs are required for optimal growth and for the development of organs including the heart. Hence, defining the optimal glucose and FA balance during fetal life is critical for normal cardiovascular development and function. Further, obesity, insulin resistance, and diabetes may co-present with CVD which will require more precise treatment based on individual treatment needs. Cardiac glucolipotoxicity should be investigated in the context of cardioprotection given that diverse populations with different genetic constitutions and environmental situations will be variably affected by a nutritional cardiac insult e.g., saturated FAs, and respond differently to a nutritional cardioprotective agent, e.g., omega-3 FAs.

## 8. Conclusions

Glucolipotoxicity induces adverse cardiac outcomes, highlighting the importance of physiological glycemia and lipidemia to maintain a healthy heart. The quality of FA shapes cardiac structure and function, often influencing survival. A healthy FA balance is therefore critical for maintaining cardiac integrity and function.

## Figures and Tables

**Table 1:** The fetal and postnatal cardiac glucose and fatty acid utilization.

	Fetal Heart	Developed Heart
Main fuel source	Glucose	Fatty acids
Glucose transport	GLUT1	GLUT4
Insulin dependency	Insulin-independent	Insulin-dependent
Meeting energy demand	Anaerobic glycolysis	Fatty acid β-oxidation
Cardiac fatty acid uptake	FAT/CD36; FATP1	FAT/CD36; FATP1
Cardiac insulin resistance:		
glucose oxidation	-	Decreased
Cardiac insulin resistance:		
fatty acid oxidation	-	Normal or increased

Cardiac glucose and fatty acid utilization in the fetal and developed heart varies in substrate and transport preference. In cardiac insulin resistance, glucose oxidation is decreased whereas fatty acid oxidation is normal or increased.

## References

[1] Robertson R.P., Harmon J., Tran P.O., Tanaka Y., Takahashi H. (2003). Glucose toxicity in β-cells: Type 2 diabetes, good radicals gone bad, and the glutathione connection. Diabetes.

[2] Brunner Y., Schvartz D., Priego-Capote F., Couté Y., Sanchez J.C. (2009). Glucotoxicity and pancreatic proteomics. J. Proteom..

[3] Pandolfi A., De Filippis E.A. (2007). Chronic hyperglycemia and nitric oxide bioavailability play a pivotal role in pro-atherogenic vascular modifications. Genes Nutr..

[4] Andreea S.I., Marieta C., Anca D. (2008). AGEs and glucose levels modulate type I and III procollagen mRNA synthesis in dermal fibroblasts cells culture. Exp. Diabetes Res..

[5] Duncan J.G. (2008). Lipotoxicity: What is the fate of fatty acids?. J. Lipid Res..

[6] Unger R.H. (2003). Lipid overload and overflow: Metabolic trauma and the metabolic syndrome. Trends Endocrinol. Metab..

[7] Guéant J.L., Elakoum R., Ziegler O., Coelho D., Feigerlova E., Daval J.L., Guéant-Rodriguez R.M. (2014). Nutritional models of foetal programming and nutrigenomic and epigenomic dysregulations of fatty acid metabolism in the liver and heart. Pflugers Arch. Eur. J. Physiol..

[8] Weir G.C., Laybutt D.R., Kaneto H., Bonner-Weir S., Sharma A. (2001). β-cell adaptation and decompensation during the progression of diabetes. Diabetes.

[9] Prentki M., Joly E., El-Assaad W., Roduit R. (2002). Malonyl-CoA signaling, lipid partitioning, and glucolipotoxicity: Role in β-cell adaptation and failure in the etiology of diabetes. Diabetes.

[10] Véret J., Bellini L., Giussani P., Ng C., Magnan C., Le Stunff H. (2014). Roles of sphingolipid metabolism in pancreatic β cell dysfunction induced by lipotoxicity. J. Clin. Med..

[11] Taegtmeyer H., Stanley W.C. (2011). Too much or not enough of a good thing? Cardiac glucolipotoxicity versus lipoprotection. J. Mol. Cell. Cardiol..

[12] Mdaki K.S., Larsen T.D., Wachal A.L., Schimelpfenig M.D., Weaver L.J., Dooyema S.D.R., Louwagie E.J., Baack M.L. (2016). Maternal high-fat diet impairs cardiac function in offspring of diabetic pregnancy through metabolic stress and mitochondrial dysfunction. AJP Heart Circ. Physiol..

[13] George L.A., Uthlaut A.B., Long N.M., Zhang L., Ma Y., Smith D.T., Nathanielsz P.W., Ford S.P. (2010). Different levels of overnutrition and weight gain during pregnancy have differential effects on fetal growth and organ development. Reprod. Biol. Endocrinol..

[14] Senyo S.E., Steinhauser M.L., Pizzimenti C.L., Yang V.K., Cai L., Wang M., Wu T.D., Guerquin-Kern J.L., Lechene C.P., Lee R.T. (2013). Mammalian heart renewal by pre-existing cardiomyocytes. Nature.

[15] Mercola M., Ruiz-Lozano P., Schneider M.D. (2011). Cardiac muscle regeneration: Lessons from development. Genes Dev..

[16] Murry C.E., Reinecke H., Pabon L.M. (2006). Regeneration gaps. Observations on stem cells and cardiac repair. J. Am. Coll. Cardiol..

[17] Morez C., Noseda M., Paiva M.A., Belian E., Schneider M.D., Stevens M.M. (2015). Enhanced efficiency of genetic programming toward cardiomyocyte creation through topographical cues. Biomaterials.

[18] Lie S., Hui M., McMillen I.C., Muhlhausler B.S., Posterino G.S., Dunn S.L., Wang K.C., Botting K.J., Morrison J.L. (2014). Exposure to rosiglitazone, a PPAR-γ agonist, in late gestation reduces the abundance of factors regulating cardiac metabolism and cardiomyocyte size in the sheep fetus. AJP Regul. Integr. Comp. Physiol..

[19] Burrell J.H., Boyn A.M., Kumarasamy V., Hsieh A., Head S.I., Lumbers E.R. (2003). Growth and maturation of cardiac myocytes in fetal sheep in the second half of gestation. Anat. Rec..

[20] Jonker S.S., Zhang L., Louey S., Giraud G.D., Thornburg K.L., Faber J.J. (2007). Myocyte enlargement, differentiation, and proliferation kinetics in the fetal sheep heart. J. Appl. Physiol..

[21] Woodcock E.A., Matkovich S.J. (2005). Cardiomyocytes structure, function and associated pathologies. Int. J. Biochem. Cell Biol..

[22] Fisher D.J., Heymann M.A., Rudolph A.M. (1980). Myocardial oxygen and carbohydrate consumption in fetal lambs in utero and in adult sheep. Am. J. Physiol..

[23] Lopaschuk G.D., Ussher J.R., Folmes C.D.L., Jaswal J.S., Stanley W.C. (2010). Myocardial fatty acid metabolism in health and disease. Physiol. Rev..

[24] Lopaschuk G.D., Jaswal J.S. (2010). Energy metabolic phenotype of the cardiomyocyte during development, differentiation, and postnatal maturation. J. Cardiovasc. Pharmacol..

[25] Gluckman P.D., Sizonenko S.V., Bassett N.S. (1999). The transition from fetus to neonate—An endocrine perspective. Acta Paediatr..

[26] Hay W.W. (1994). Placental transport of nutrients to the fetus. Horm. Res..

[27] Lopaschuk G.D., Spafford M.A., Marsh D.R. (1991). Glycolysis is predominant source of myocardial ATP production immediately after birth. Am. J. Physiol..

[28] Stanley W.C., Recchia F.A., Lopaschuk G.D. (2005). Myocardial substrate metabolism in the normal and failing heart. Physiol. Rev..

[29] Ferrari R., Cargnoni A., Ceconi C. (2006). Anti-ischaemic effect of ivabradine. Pharmacol. Res..

[30] Beauchamp B., Thrush A., Quizi J., Antoun G., McIntosh N., Al-dirbashi O., Patti M.-E., Harper M.-E. (2015). Undernutrition during pregnancy in mice leads to dysfunctional cardiac muscle respiration in adult offspring. Biosci. Rep..

[31] Razeghi P., Young M.E., Alcorn J.L., Moravec C.S., Frazier O.H., Taegtmeyer H. (2001). Metabolic gene expression in fetal and failing human heart. Circulation.

[32] Sack M.N., Rader T.A., Park S., Bastin J., McCune S.A., Kelly D.P. (1996). Fatty acid oxidation enzyme gene expression is downregulated in the failing heart. Circulation.

[33] Doenst T., Pytel G., Schrepper A., Amorim P., Färber G., Shingu Y., Mohr F.W., Schwarzer M. (2010). Decreased rates of substrate oxidation ex vivo predict the onset of heart failure and contractile dysfunction in rats with pressure overload. Cardiovasc. Res..

[34] Sharov V.G., Todor A.V., Silverman N., Goldstein S., Sabbah H.N. (2000). Abnormal mitochondrial respiration in failed human myocardium. J. Mol. Cell. Cardiol..

[35] Sharov V., Goussev A., Lesch M., Goldstein S., Sabbah H. (1998). Abnormal mitochondrial function in myocardium of dogs with chronic heart failure. J. Mol. Cell. Cardiol..

[36] Anderson E.J., Kypson A.P., Rodriguez E., Anderson C.A., Lehr E.J., Neufer P.D. (2009). Substrate-specific derangements in mitochondrial metabolism and redox balance in the atrium of the type 2 diabetic human heart. J. Am. Coll. Cardiol..

[37] Boudina S., Sena S., Theobald H., Sheng X., Wright J.J., Xia X.H., Aziz S., Johnson J.I., Bugger H., Zaha V.G. (2007). Mitochondrial energetics in the heart in obesity-related diabetes: Direct evidence for increased uncoupled respiration and activation of uncoupling proteins. Diabetes.

[38] Aguer C., McCoin C.S., Knotts T.A., Thrush A.B., Ono-Moore K., McPherson R., Dent R., Hwang D.H., Adams S.H., Harper M.-E. (2015). Acylcarnitines: Potential implications for skeletal muscle insulin resistance. FASEB J..

[39] Grimsgaard S., Bønaa K.H., Jacobsen B.K., Bjerve K.S. (1999). Plasma saturated and linoleic fatty acids are independently associated with blood pressure. Hypertension.

[40] Tamaya-Mori N., Uemura K., Iguchi A. (2002). Gender differences in the dietary lard-induced increase in blood pressure in rats. Hypertension.

[41] Hui R.M., Robillard J.H., Grose M., Lebel P.F. (1991). Arachidonic acid does not share the antihypertensive properties of linoleic acid and fish oilomega-3 fatty acids in a model of angiotensin II-induced hypertension in the rat. Clin. Investig. Med..

[42] Sellmayer A., Danesch U., Weber P.C. (1996). Effects of different polyunsaturated fatty acids on growth-related early gene expression and cell growth. Lipids.

[43] Földes G., Vajda S., Lakó-Futó Z., Sármán B., Skoumal R., Ilves M., de Châtel R., Karádi I., Tóth M., Ruskoaho H. (2006). Distinct modulation of angiotensin II-induced early left ventricular hypertrophic gene programming by dietary fat type. J. Lipid Res..

[44] Bugger H., Abel E.D. (2014). Molecular mechanisms of diabetic cardiomyopathy. Diabetologia.

[45] Fuentes-Antrás J., Picatoste B., Ramírez E., Egido J., Tuñón J., Lorenzo Ó. (2015). Targeting metabolic disturbance in the diabetic heart. Cardiovasc. Diabetol..

[46] Goldberg I.J., Trent C.M., Schulze P.C. (2012). Lipid metabolism and toxicity in the heart. Cell Metab..

[47] Duncan J.G. (2011). Mitochondrial dysfunction in diabetic cardiomyopathy. Biochim. Biophys. Acta.

[48] Wallace D.C. (2011). Bioenergetic origins of complexity and disease. Cold Spring Harb. Symp. Quant. Biol..

[49] Costantino S., Paneni F., Cosentino F. (2015). Ageing, metabolism and cardiovascular disease. J. Physiol..

[50] Grynberg A., Demaison L. (1996). Fatty acid oxidation in the heart. J. Cardiovasc. Pharmacol..

[51] Makinde A.O., Kantor P.F., Lopaschuk G.D. (1998). Maturation of fatty acid and carbohydrate metabolism in the newborn heart. Mol. Cell. Biochem..

[52] Hue L., Taegtmeyer H. (2009). The Randle cycle revisited: A new head for an old hat. AJP Endocrinol. Metab..

[53] Hu F.B., Manson J.E., Willett W.C. (2001). Types of dietary fat and risk of coronary heart disease: A critical review. J. Am. Coll. Nutr..

[54] Simopoulos A.P. (1999). Essential fatty acids in health and chronic disease. Am. J. Clin. Nutr..

[55] Taegtmeyer H., Overturf M.L. (1988). Effects of moderate hypertension on cardiac function and metabolism in the rabbit. Hypertension.

[56] Kagaya Y., Kanno Y., Takeyama D., Ishide N., Maruyama Y., Takahashi T., Ido T., Takishima T. (1990). Effects of long-term pressure overload on regional myocardial glucose and free fatty acid uptake in rats. Circulation.

[57] Zahabi A., Deschepper C.F. (2001). Long-chain fatty acids modify hypertrophic responses of cultured primary neonatal cardiomyocytes. J. Lipid Res..

[58] Aguila M.B., Alberto Mandarim-de-Lacerda C.A. (2001). Blood pressure, ventricular volume and number of cardiomyocyte nuclei in rats fed for 12 months on diets differing in fat composition. Mech. Ageing Dev..

[59] Carroll J.F., Braden D.S., Cockrell K., Mizelle H.L. (1997). Obese hypertensive rabbits develop concentric and eccentric hypertrophy and diastolic filling abnormalities. Am. J. Hypertens..

[60] Chu K.C., Sohal R.S., Sun S.C., Burch G.E., Colcolough H.L. (1969). Lipid cardiomyopathy of the hypertrophied heart of goldthioglucose obese mice. J. Pathol..

[61] Fitzgerald S.M., Henegar J.R., Brands M.W., Henegar L.K., Hall J.E. (2001). Cardiovascular and renal responses to a high-fat diet in Osborne-Mendel rats. AJP Regul. Integr. Comp. Physiol..

[62] Sundström J., Lind L., Vessby B., Andrén B., Aro A., Lithell H. (2001). Dyslipidemia and an unfavorable fatty acid profile predict left ventricular hypertrophy 20 years later. Circulation.

[63] Taniguchi C.M., Emanuelli B., Kahn C.R. (2006). Critical nodes in signalling pathways: Insights into insulin action. Nat. Rev. Mol. Cell Biol..

[64] Catalano P.M., Presley L., Minium J., Hauguel-de Mouzon S. (2009). Fetuses of obese mothers develop insulin resistance in utero. Diabetes Care.

[65] Gray S., Kim J.K. (2011). New insights into insulin resistance in the diabetic heart. Trends Endocrinol. Metab..

[66] Bell D.S. (2003). Heart failure: The frequent, forgotten, and often fatal complication of diabetes. Diabetes Care.

[67] Wright C.S., Rifas-Shiman S.L., Rich-Edwards J.W., Taveras E.M., Gillman M.W., Oken E. (2009). Intrauterine exposure to gestational diabetes, child adiposity, and blood pressure. Am. J. Hypertens..

[68] Shimizu I., Minamino T., Toko H., Okada S., Ikeda H., Yasuda N., Tateno K., Moriya J., Yokoyama M., Nojima A. (2010). Excessive cardiac insulin signaling exacerbates systolic dysfunction induced by pressure overload in rodents. J. Clin. Investig..

[69] Boden G. (2011). Obesity, insulin resistance and free fatty acids. Curr. Opin. Endocrinol. Diabetes Obes..

[70] Reaven G.M. (1988). Role of insulin resistance in human disease. Diabetes.

[71] Park S.Y., Cho Y.R., Finck B.N., Kim H.J., Higashimori T., Hong E.G., Lee M.K., Danton C., Deshmukh S., Cline G.W. (2005). Cardiac-specific overexpression of peroxisome proliferator-activated receptor-α causes insulin resistance in heart and liver. Diabetes.

[72] Abel E.D., O’Shea K.M., Ramasamy R. (2012). Insulin resistance: Metabolic mechanisms and consequences in the heart. Arterioscler. Thromb. Vasc. Biol..

[73] Covarrubias L., Hernández-García D., Schnabel D., Salas-Vidal E., Castro-Obregón S. (2008). Function of reactive oxygen species during animal development: Passive or active?. Dev. Biol..

[74] Franco Mdo C., Dantas A.P., Akamine E.H., Kawamoto E.M., Fortes Z.B., Scavone C., Tostes R.C., Carvalho M.H., Nigro D. (2002). Enhanced oxidative stress as a potential mechanism underlying the programming of hypertension in utero. J. Cardiovasc. Pharmacol..

[75] Du Toit E.F., Donner D.G., Arora S. (2012). Myocardial insulin resistance: An overview of its causes, effects, and potential therapy. Insulin Resistance.

[76] Stahl A., Gimeno R.E., Tartaglia L.A., Lodish H.F. (2001). Fatty acid transport proteins: A current view of a growing family. Trends Endocrinol. Metab..

[77] Park S.H., Gammon S.R., Knippers J.D., Paulsen S.R., Rubink D.S., Winder W.W. (2002). Phosphorylation-activity relationships of AMPK and acetyl-CoA carboxylase in muscle. J. Appl. Physiol..

[78] Piñeiro R., Iglesias M.J., Gallego R., Raghay K., Eiras S., Rubio J., Diéguez C., Gualillo O., González-Juanatey J.R., Lago F. (2005). Adiponectin is synthesized and secreted by human and murine cardiomyocytes. FEBS Lett..

[79] Lopaschuk G.D., Gamble J. (1994). Acetyl-CoA carboxylase: An important regulator of fatty acid oxidation in the heart. Can. J. Physiol. Pharmacol..

[80] Vega R.B., Huss J.M., Kelly D.P. (2000). The coactivator PGC-1 cooperates with peroxisome proliferator-activated receptor alpha in transcriptional control of nuclear genes encoding mitochondrial fatty acid oxidation enzymes. Mol. Cell. Biol..

[81] Sugden M.C., Holness M.J. (2006). Mechanisms underlying regulation of the expression and activities of the mammalian pyruvate dehydrogenase kinases. Arch. Physiol. Biochem..

[82] Wang K.C.W., Brooks D.A., Thornburg K.L., Morrison J.L. (2012). Activation of IGF-2R stimulates cardiomyocyte hypertrophy in the late gestation sheep fetus. J. Physiol..

[83] Nishikimi T., Maeda N., Matsuoka H. (2006). The role of natriuretic peptides in cardioprotection. Cardiovasc. Res..

[84] Kelly D.P., Strauss A.W. (1994). Inherited cardiomyopathies. N. Engl. J. Med..

[85] Brookheart R.T., Michel C.I., Schaffer J.E. (2009). As a matter of fat. Cell Metab..

[86] Petrie J.R., Guzik T.J., Touyz R. (2018). Diabetes, hypertension, and cardiovascular disease: Clinical insights and vascular mechanisms. Can. J. Cardiol..

[87] An D., Rodrigues B. (2006). Role of changes in cardiac metabolism in development of diabetic cardiomyopathy. AJP Heart Circ. Physiol..

[88] Boudina S., Abel E.D. (2010). Diabetic cardiomyopathy, causes and effects. Rev. Endocr. Metab. Disord..

[89] Kannel W.B., Hjortland M., Castelli W.P. (1974). Role of diabetes in congestive heart failure: The Framingham study. Am. J. Cardiol..

[90] De Simone G., Devereux R.B., Chinali M., Lee E.T., Galloway J.M., Barac A., Panza J.A., Howard B.V. (2010). Diabetes and incident heart failure in hypertensive and normotensive participants of the Strong Heart Study. J. Hypertens..

[91] Basu R., Oudit G.Y., Wang X., Zhang L., Ussher J.R., Lopaschuk G.D., Kassiri Z. (2009). Type 1 diabetic cardiomyopathy in the Akita (Ins2WT/C96Y) mouse model is characterized by lipotoxicity and diastolic dysfunction with preserved systolic function. AJP Heart Circ. Physiol..

[92] Abe T., Ohga Y., Tabayashi N., Kobayashi S., Sakata S., Misawa H., Tsuji T., Kohzuki H., Suga H., Taniguchi S. (2002). Left ventricular diastolic dysfunction in type 2 diabetes mellitus model rats. AJP Heart Circ. Physiol..

[93] Poornima I.G., Parikh P., Shannon R.P. (2006). Diabetic cardiomyopathy: The search for a unifying hypothesis. Circ. Res..

[94] Pulinilkunnil T., Kienesberger P.C., Nagendran J., Sharma N., Young M.E., Dyck J.R.B. (2014). Cardiac-specific adipose triglyceride lipase overexpression protects from cardiac steatosis and dilated cardiomyopathy following diet-induced obesity. Int. J. Obes..

[95] Pulinilkunnil T., Kienesberger P.C., Nagendran J., Waller T.J., Young M.E., Kershaw E.E., Korbutt G., Haemmerle G., Zechner R., Dyck J.R.B. (2013). Myocardial adipose triglyceride lipase overexpression protects diabetic mice from the development of lipotoxic cardiomyopathy. Diabetes.

[96] Li C.J., Lv L., Li H., Yu D.M. (2012). Cardiac fibrosis and dysfunction in experimental diabetic cardiomyopathy are ameliorated by alpha-lipoic acid. Cardiovasc. Diabetol..

[97] Wakasaki H., Koya D., Schoen F.J., Jirousek M.R., Ways D.K., Hoit B.D., Walsh R.A., King G.L. (1997). Targeted overexpression of protein kinase C 2 isoform in myocardium causes cardiomyopathy. Proc. Natl. Acad. Sci. USA.

[98] Buchanan J., Mazumder P.K., Hu P., Chakrabarti G., Roberts M.W., Ui J.Y., Cooksey R.C., Litwin S.E., Abel E.D. (2005). Reduced cardiac efficiency and altered substrate metabolism precedes the onset of hyperglycemia and contractile dysfunction in two mouse models of insulin resistance and obesity. Endocrinology.

[99] Ghosh S., An D., Pulinilkunnil T., Qi D., Lau H.C.S., Abrahani A., Innis S.M., Rodrigues B. (2004). Role of dietary fatty acids and acute hyperglycemia in modulating cardiac cell death. Nutrition.

[100] Trivedi P.C., Bartlett J.J., Perez L.J., Brunt K.R., Legare J.F., Hassan A., Kienesberger P.C., Pulinilkunnil T. (2016). Glucolipotoxicity diminishes cardiomyocyte TFEB and inhibits lysosomal autophagy during obesity and diabetes. Biochim. Biophys. Acta.

[101] Van De Weijer T., Schrauwen-Hinderling V.B., Schrauwen P. (2011). Lipotoxicity in type 2 diabetic cardiomyopathy. Cardiovasc. Res..

[102] Kim J.W., Yoon K.H. (2011). Glucolipotoxicity in pancreatic β-cells. Diabetes Metab. J..

[103] Xia J.Y., Lloyd-Jones D.M., Khan S.S. (2018). Association of body mass index with mortality in cardiovascular disease: New insights into the obesity paradox from multiple perspectives. Trends Cardiovasc. Med..

[104] Lavie C.J., Milani R.V., Ventura H.O. (2009). Obesity and cardiovascular disease: Risk factor, paradox, and impact of weight loss. J. Am. Coll. Cardiol..

[105] Nettleton J.A., Brouwer I.A., Geleijnse J.M., Hornstra G. (2017). Saturated fat consumption and risk of coronary heart disease and ischemic stroke: A science update. Ann. Nutr. Metab..

[106] Mozaffarian D., Benjamin E.J., Go A.S., Arnett D.K., Blaha M.J., Cushman M., De Ferranti S., Després J.P., Fullerton H.J., Howard V.J. (2015). Heart disease and stroke statistics-2015 update: A report from the American Heart Association Statistics Committee and Stroke Statistics Subcommittee. Circulation.

[107] Hooper L., Martin N., Abdelhamid A., Davey Smith G. (2015). Reduction in saturated fat intake for cardiovascular disease. Cochrane Database Syst. Rev..

[108] Wang D.D., Li Y., Chiuve S.E., Stampfer M.J., Manson J.A.E., Rimm E.B., Willett W.C., Hu F.B. (2016). Association of specific dietary fats with total and cause-specific mortality. JAMA Intern. Med..

[109] Duda M.K., O’Shea K.M., Stanley W.C. (2009). Omega-3 polyunsaturated fatty acid supplementation for the treatment of heart failure: Mechanisms and clinical potential. Cardiovasc. Res..

[110] Cerf M.E., Louw J. (2014). Islet cell response to high fat programming in neonate, weanling and adolescent Wistar rats. JOP.

[111] Gittes G.K. (2009). Developmental biology of the pancreas: A comprehensive review. Dev. Biol..

[112] Miralles F. (1998). TGF-beta plays a key role in morphogenesis of the pancreatic islets of Langerhans by controlling the activity of the matrix metalloproteinase MMP-2. J. Cell Biol..

[113] Dyntar D., Eppenberger-Eberhardt M., Maedler K., Pruschy M., Eppenberger H.M., Spinas G.A., Donath M.Y. (2001). Glucose and palmitic acid induce degeneration of myofibrils and modulate apoptosis in rat adult cardiomyocytes. Diabetes.

[114] Listenberger L.L., Ory D.S., Schaffer J.E. (2001). Palmitate-induced apoptosis can occur through a ceramide-independent pathway. J. Biol. Chem..

[115] Fiordaliso F., Leri A., Cesselli D., Limana F., Safai B., Nadal-Ginard B., Anversa P., Kajstura J. (2001). Hyperglycemia activates p53 and p53-regulated genes leading to myocyte cell death. Diabetes.

[116] Wang H.J., Lee E.Y., Han S.J., Kim S.H., Lee B.W., Ahn C.W., Cha B.S., Lee H.C. (2012). Dual pathways of p53 mediated glucolipotoxicity-induced apoptosis of rat cardiomyoblast cell: Activation of p53 pro-apoptosis and inhibition of Nrf2-NQO1 anti-apoptosis. Metabolism.

[117] Kim W.H., Lee J.W., Suh Y.H., Lee H.J., Lee S.H., Oh Y.K., Gao B., Jung M.H. (2007). AICAR potentiates ROS production induced by chronic high glucose: Roles of AMPK in pancreatic β-cell apoptosis. Cell. Signal..

[118] Cai L., Li W., Wang G., Guo L., Jiang Y., James Kang Y. (2002). Hyperglycemia-induced apoptosis in mouse myocardium: Mitochondrial cytochrome c-mediated caspase-3 activation pathway. Diabetes.

[119] Sharma S., Adrogue J.V., Golfman L., Uray I., Lemm J., Youker K., Noon G.P., Frazier O.H., Taegtmeyer H. (2004). Intramyocardial lipid accumulation in the failing human heart resembles the lipotoxic rat heart. FASEB J..

[120] Boudina S. (2006). Mitochondrial uncoupling: A key contributor to reduced cardiac efficiency in diabetes. Physiology.

[121] González-Rodríguez A., Mayoral R., Agra N., Valdecantos M.P., Pardo V., Miquilena-Colina M.E., Vargas-Castrillón J., Lo Iacono O., Corazzari M., Fimia G.M. (2014). Impaired autophagic flux is associated with increased endoplasmic reticulum stress during the development of NAFLD. Cell Death Dis..

[122] Karunakaran U., Kim H.J., Kim J.Y., Lee I.K. (2012). Guards and culprits in the endoplasmic reticulum: Glucolipotoxicity and β-cell failure in type II diabetes. Exp. Diabetes Res..

[123] Mizushima N., Yoshimori T., Ohsumi Y. (2011). The role of Atg proteins in autophagosome formation. Annu. Rev. Cell Dev. Biol..

[124] Lippai M., Low P. (2014). The role of the selective adaptor p62 and ubiquitin-like proteins in autophagy. BioMed Res. Int..

[125] Casas S., Gomis R., Gribble F.M., Altirriba J., Knuutila S., Novials A. (2007). Impairment of the ubiquitin-proteasome pathway is a downstream endoplasmic reticulum stress response induced by extracellular human islet amyloid polypeptide and contributes to pancreatic beta-cell apoptosis. Diabetes.

[126] Tsukamoto O., Minamino T., Okada K.I., Shintani Y., Takashima S., Kato H., Liao Y., Okazaki H., Asai M., Hirata A. (2006). Depression of proteasome activities during the progression of cardiac dysfunction in pressure-overloaded heart of mice. Biochem. Biophys. Res. Commun..

[127] Vlassara H., Uribarri J. (2014). Advanced glycation end products (AGE) and diabetes: Cause, effect, or both?. Curr. Diab. Rep..

[128] Sedeek M., Montezano A.C., Hebert R.L., Gray S.P., Di Marco E., Jha J.C., Cooper M.E., Jandeleit-Dahm K., Schiffrin E.L., Wilkinson-Berka J.L. (2012). Oxidative stress, nox isoforms and complications of diabetes-potential targets for novel therapies. J. Cardiovasc. Transl. Res..

